# Identification of Prognostic Immune-Related Genes in Pancreatic Adenocarcinoma and Establishment of a Prognostic Nomogram: A Bioinformatic Study

**DOI:** 10.1155/2020/1346045

**Published:** 2020-06-09

**Authors:** Guolin Wu, Zhenfeng Deng, Zongrui Jin, Jilong Wang, Banghao Xu, Jingjing Zeng, Minhao Peng, Zhang Wen, Ya Guo

**Affiliations:** ^1^Department of Hepatobiliary Surgery, The First Affiliated Hospital of Guangxi Medical University, Nanning 530021, China; ^2^Department of Pathology, The First Affiliated Hospital of Guangxi Medical University, Nanning 530021, China

## Abstract

**Background:**

The prognosis of pancreatic adenocarcinoma (PAAD) is extremely poor and has not been improved. Thus, an effective method to assess the prognosis of patients must be established to improve their survival rate.

**Method:**

This study investigated immune-related genes that could be used as potential therapeutic targets for PAAD. Level 3 gene expression data from the PAAD cohort and the relevant clinical information were obtained from The Cancer Genome Atlas (TCGA) database. For validation, other PAAD datasets (DSE62452) were downloaded from the Gene Expression Omnibus (GEO) database. The PAAD datasets from TCGA and GEO were used to screen immune-related genes through the Molecular Signatures Database using gene set enrichment analysis. Then, the overlapping immune-related genes of the two datasets were identified. Coexpression networks of the immune-related genes were constructed.

**Results:**

A signature of three immune-related genes (CKLF, ERAP2, and EREG) was identified in patients with PAAD. The signature could be used to divide the patients with PAAD into high- and low-risk groups based on their median risk score. Multivariate Cox regression analysis was performed to determine the independent prognostic factors of PAAD. Time-dependent receiver operating characteristic (ROC) curve analysis was conducted to assess the prediction accuracy of the prognostic signature. Last, a nomogram was established to assess the individualized prognosis prediction model based on the clinical characteristics and risk score of the TCGA PAAD dataset. The accuracy of the prognostic signature was further evaluated through functional evaluation and principal component analysis.

**Conclusions:**

The results indicated that the signature of three immune-related genes had excellent predictive value for PAAD. These findings might help improve personalized treatment and medical decisions.

## 1. Introduction

Pancreatic cancer is a leading cause of death in developed countries, and it is a common malignant tumor worldwide [1]. The main tumor type of pancreatic cancer is pancreatic adenocarcinoma (PAAD), which accounts for approximately 85% of cases [2]. The prognosis of pancreatic cancer is extremely poor, and the 5-year survival rate is estimated to be less than 5% [1]. Pancreatic cancer is expected to surpass breast cancer and become the third leading cause of cancer death [3]. Studies have predicted that the mortality rate of pancreatic cancer in malignant tumors worldwide will have ranked second by 2030 [4]. An important reason for the low survival rate of pancreatic cancer is that most patients are diagnosed at the end stage of the disease [5]. Smoking, high-fat diet, obesity, alcoholism, diabetes, and chronic pancreatitis are risk factors for pancreatic cancer [6–8]. The pathogenesis of familial pancreatic cancer is closely related to *CDKN2A*, *BRCA1*, *BRCA2*, and *PALB2* [9]. The only possible treatment for pancreatic cancer is surgical resection, but only a few patients with early-stage pancreatic cancer are eligible to undergo resection [10]. Chemotherapy, targeted therapy, and immunotherapy for pancreatic cancer treatment improve a patient's survival time [11]. Immunotherapy has become a new pillar of cancer treatment for more than a decade, and it has offered new hope for reducing the morbidity and mortality of this refractory disease [12]. The development of immunotherapy for PAAD treatment faces challenges because of the poor immunogenic nature of PAAD [13]. Nevertheless, a large percentage of patients with PAAD may benefit from immunotherapy in the future [14]. With remarkable progress in bioinformatics, prognostic gene expression characteristics have been extensively developed for PAAD [15]. The development of tumor molecular biology has further promoted tumor therapy based on immune-related genes. Therefore, the abnormal expression of immune-related genes may have prognostic value for patients with PAAD and provide a new basis for administering tumor immunotherapy for PAAD. In this study, immune-related genes associated with the prognosis of PAAD were identified on the basis of RNA-seq data from TCGA through the Molecular Signatures Database (MSigDB) [16], and a risk score model for PAAD prognosis was constructed. A prognostic nomogram that combined prognostic gene trait risk models and clinical prognostic factors was established to predict overall survival (OS). The reliability of this method was verified through the GEO database.

## 2. Materials and Methods

### 2.1. Datasets Source

The level 3 gene expression data from the PAAD cohort and corresponding clinical information were obtained from the data portal of TCGA (https://portal.gdc.cancer.gov/, accessed October 11, 2019). Relevant clinical information, such as gender, age, radical resection, grade, alcohol history, survival, and outcome, was also obtained from the data portal of TCGA. For validation, gene expression microarray datasets (DSE62452) were downloaded from the Gene Expression Omnibus (GEO, https://http://www.ncbi.nlm. https://nih.gov/geo/). The data used in this study were downloaded from GEO and TCGA, so data were acquired and applied in accordance with GEO and TCGA publishing guidelines and data access policies. Therefore, no additional approval from an ethics committee was required.

### 2.2. Immune-Related Gene Screening

A list of immune-related genes was extracted from the datasets of immune system process (M13664) and immune response (M19817) from MSigDB (http://software.broadinstitute.org/gsea/msigdb/index.jsp) [16]. The expression data of these genes were screened from the PAAD cases of TCGA and GEO. Immune-related prognostic genes were further evaluated with univariate Cox proportional hazard regression by using a “survival” package (version 2.44-1.1) on the R platform (version 3.6.1). Genes with *P* < 0.05 and |hazard ratio (HR)| >1.00 were considered to be prognostic risk genes, and their expression levels were significantly associated with OS in PAAD.

### 2.3. Signature Development

The prognostic immune-related genes were analyzed using multivariate Cox regression analysis with OS as the dependent variable to evaluate their roles in predicting PAAD survival. A prognostic risk score model was prepared via the linear combination of the expression levels of immune-related genes with the multivariate Cox regression coefficient (*β*) as the weight [17]. The risk scores were calculated using the prognostic gene signatures. The risk score formula was as follows: risk score = expression of gene1 × *β*1 + expression of gene2 × *β*2 + ⋯expression of gene n × *β*n [18, 19]. A total of 142 cases were divided into high- and low-risk groups based on the median risk score. |HR| >1.0 and *P* < 0.05 were selected among the TCGA and GEO datasets as a cut-off. Then, three genes were chosen for signature development. A receiver operating characteristic (ROC) curve was established over time on the R platform to assess the accuracy of the risk score model for predicting the prognosis of PAAD [20].

### 2.4. Functional and Pathway Enrichment Analyses

The functional enrichment analyses of the immune-related genes mainly involving Gene Ontology (GO) terms and Kyoto Encyclopedia of Genes and Genomes (KEGG) pathway analysis were carried out using the “cluster Profiler” R package [21]. GO analysis revealed the functions of the immune-related genes in biological processes (BP), cellular components (CC), and molecular functions (MF), and the KEGG analysis showed the pathway enrichment of the immune-related genes. *P* < 0.05 was considered statistically significant.

### 2.5. Predictive Nomogram Construction and Validation

A prognostic signature based on the expression of immune-related genes was comprehensively analyzed to further assess the prognostic model. After collinearity was tested, a Norman diagram was predicted using a stepwise Cox regression model to predict the 1-, 3-, and 5-year OS rates of the patients with PAAD the datasets from TCGA and GEO. Kaplan-Meier analysis and area under the curve (AUC) comparison of the ROC curve were applied to predict and observe the OS rate for assessing the performance of the prognostic nomogram. We not only compared the clinical outcomes of the low-risk and high-risk groups but also evaluated the prognostic value of PAAD with a risk score through a nomogram. The potential application of risk scores in the prediction of clinical status was also explored.

### 2.6. Statistical Analysis (OS Curve)

Kaplan-Meier survival analysis by log-rank test was conducted to identify the immune-related genes associated with the prognosis of PAAD. Univariate, multivariate, and Cox regression analyses and principal component analysis (PCA) were performed in R and SPSS version 22.0 (Chicago, IL, USA). Univariate and multivariate Cox regression analyses as well as performed to assess survival. HRs and 95% confidence intervals (CIs) were calculated to identify OS-associated genes. Statistical significance was set at *P* < 0.05.

## 3. Results

### 3.1. Identification of Immune-Related Genes with Prognostic Value

**Figure 1:**
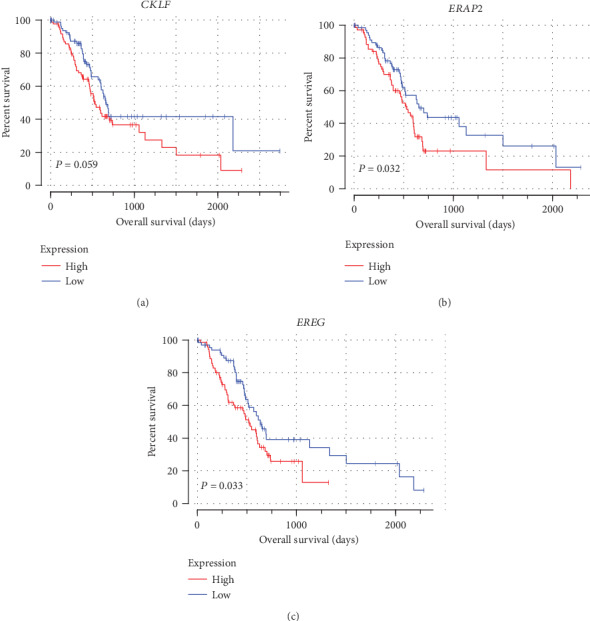
Kaplan-Meier curves of three prognostic immune-related genes in PAAD. The order is as follows: (a) *CKLF*, *P* = 0.059; (b) *ERAP2*, *P* = 0.032; and (c) *EREG*, *P* = 0.033.

The clinical information and gene expression profiles of 142 PAAD cases were downloaded from the database from TCGA for further analysis. A total of 332 immune-related genes were selected from MSigDB v4.0 [16] (immune system process, immune response); (http://www.broadinstitute.org/gsea/msigdb/index.jsp). For validation, gene expression microarray datasets (DSE62452; https://www.ncbi.nlm.nih.gov/geo/query/acc.cgi?acc=GSE62452) were downloaded from the GEO dataset. Immune-related genes were screened in the same way. Immune-related prognostic genes were further evaluated via univariate Cox proportional hazard regression by using the survival software package. Coexpression networks of the identified immune-related genes were constructed. Then, three genes (chemokine-like factor [*CKLF*], endoplasmic reticulum aminopeptidase 2 [*ERAP2*], and epiregulin [*EREG*]) that indicated a high risk in the TCGA, and GEO databases were highlighted (Table S1). The Kaplan-Meier analysis results of *CKLF*, *ERAP2*, and *EREG* are shown in Figure 1.

### 3.2. Prognostic Model Construction and ROC Curve Analysis

**Figure 2:**
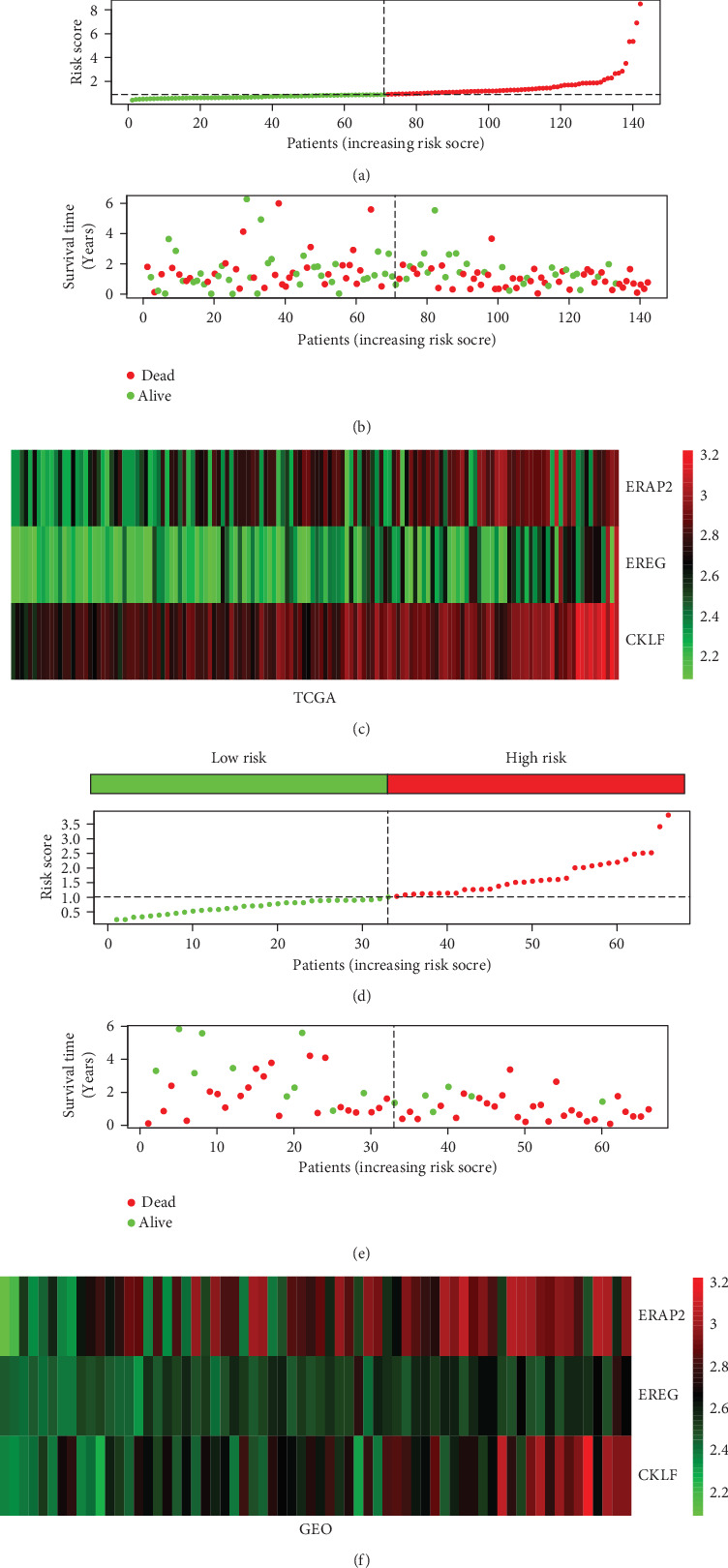
Prognostic risk score model analysis of three prognostic immune genes in PAAD patients. In the database from TCGA, from top to bottom are (a) the risk score, (b) patient survival status distribution, and (c) three hub gene expression heat maps for the low- and high-risk groups. In the GEO database, from top to bottom are (d) the risk score, (e) patient survival status distribution, and (f) three hub gene expression heat maps for the low- and high-risk groups.

**Figure 3:**
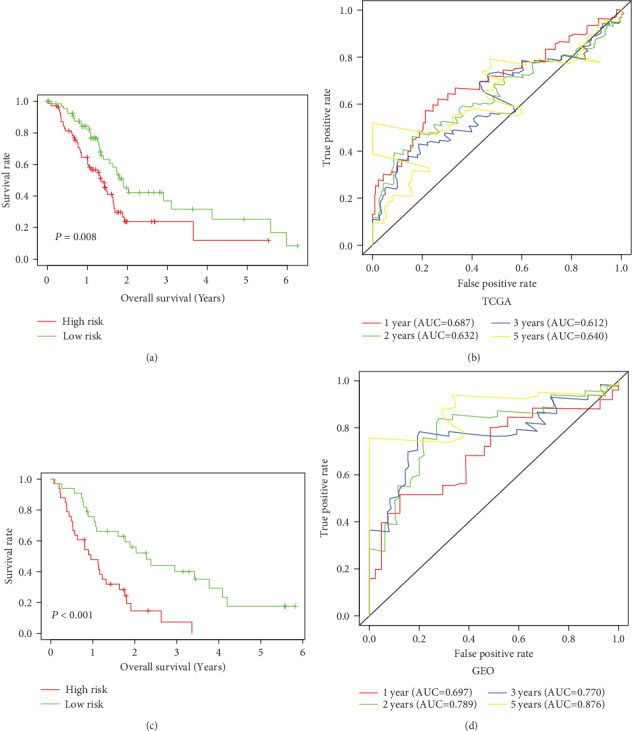
Kaplan-Meier curves for low-risk and high-risk populations in different databases. ROC curve for predicting survival in PAAD patients by the risk score (a) Kaplan-Meier curves for low- and high-risk groups using the database from TCGA (*P* = 0.008). (b) The ROC curve for predicting the survival rate of PAAD based on the risk score of the TCGA database. (c) Kaplan-Meier curves for low- and high-risk groups in GEO (*P* < 0.001). (d) The ROC curve for predicting the survival rate of PAAD based on the risk score of the GEO database.

A signature of three immune-related genes was developed using a risk score method [18, 19]. The risk score formula was as follows: risk score = expression of CKLF × (0.9452) + expression of ERAP2 × (0.2968) + expression of EREG × (0.3896). The 142 patients in the database from TCGA were divided into high- and low-risk groups based on their median risk score. Survival analysis showed that the OS of the patients in the high-risk group was shorter (high risk and low risk: 486 vs. 691 days) than it was in the low-risk group. The risk of death significantly increased in the patients with high-risk scores (*P* value = 0.009; HR = 1.852; 95%CI = 1.165–2.944; Table 1, Figures 2(a) and 2(b)). In addition, the risk heat maps of the gene expression profiles of *CKLF*, *ERAP*2, and *EREG* indicated that the expression levels of these genes were higher in the high-risk group than in the low-risk group (Figure 2(c)). Validation by the GEO database further confirmed that the survival rate of the high-risk score group was lower than that of the low-risk group (Figures 2(d)–2(f)). The Kaplan-Meier curves of the OS rates of the patients with PAAD from the different groups were stratified in terms of the signature from the TCGA and GEO datasets. In the database from TCGA, the OS of the low-risk group was longer than that of the high-risk group (*P* = 0.008, Figure 3(a)). The same result was obtained from the GEO database (*P* < 0.001, Figure 3(c)). Time-dependent ROC curve analysis was carried out to assess the prediction accuracy of the prognostic signature. Our results showed that the prognostic signature from the database from TCGA in the current study performed well in predicting 1-, 2-, and 3-year survival rates. The area under the curve values for 1-, 2-, and 3-year survival were 0.687, 0.632, and 0.612, respectively (Figure 3(b)). The prognostic signatures of the GEO database also performed well in predicting 1-, 2-, and 3-year survival rates (Figure 3(d)).

### 3.3. Predictive Nomogram Construction and Validation

**Figure 4:**
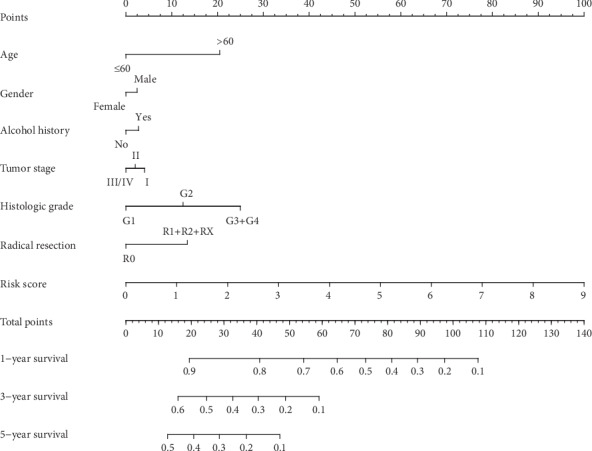
The relationship between risk score and clinical information. A prognostic nomogram predicting 1-, 3-, and 5-year OS of PAAD in the database from TCGA.

A comprehensive nomogram survival analysis was conducted to investigate the relationship between the risk scores and clinical characteristics of OS for patients with PAAD. A nomogram was drawn with RMS and its auxiliary packages based on the clinical information of PAAD and the risk score. The results confirmed that the prognostic markers of the risk score significantly influenced the risk points, whereas other clinical features had a lower effect on the risk points (Figure 4). In our nomogram, the shortcoming was that the prognostic signature of tumor stage could not perform well in PAAD.

### 3.4. Low- and High-Risk Groups Displayed Different Immune Status

**Figure 5:**
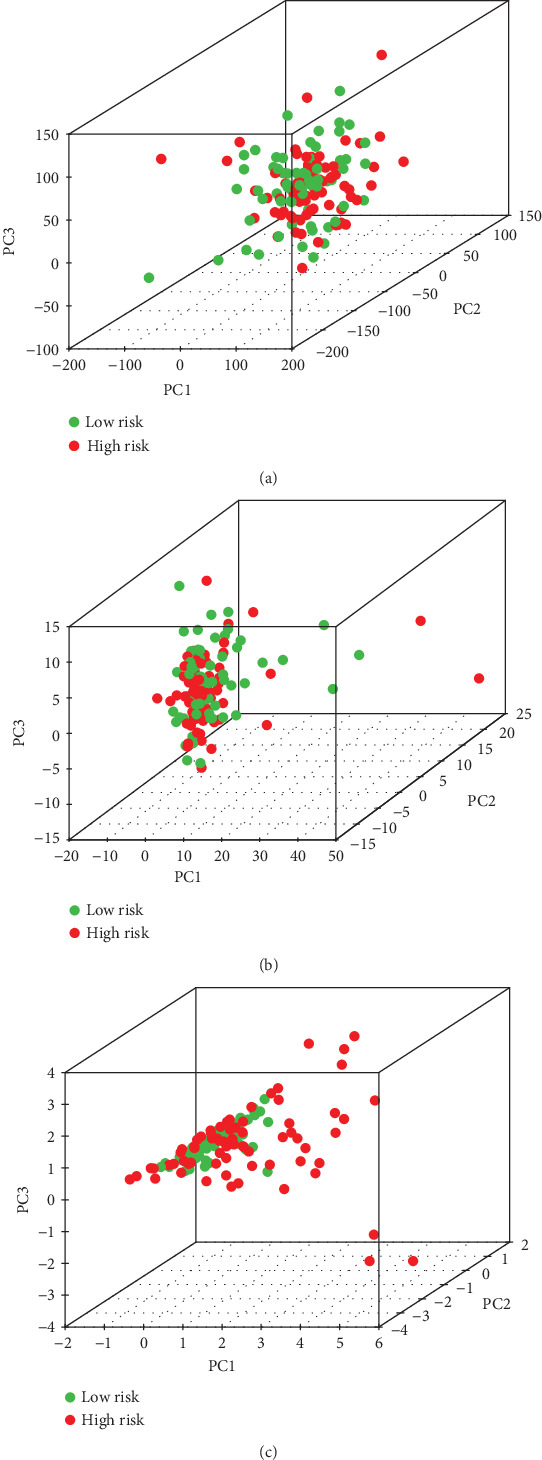
Principal component analysis between low-risk and high-risk groups based on different classification methods. (a) All genes. (b) Immune genes. (c) Risk genes.

PCA was performed to study the differences between low- and high-risk populations based on the expression profiles of all genes, immune-related genes, and risk-related genes (Figures 5(a)–5(c)). Our results indicated that low- and high-risk groups were usually distributed in different directions. According to the prognostic signature of immune-related genes, patients in the high-risk group could be clearly distinguished from patients in the low-risk group. Therefore, the immune status of PAAD with a specific gene signature was different from other genes.

### 3.5. Functional Enrichment Analysis of Genes

**Figure 6:**
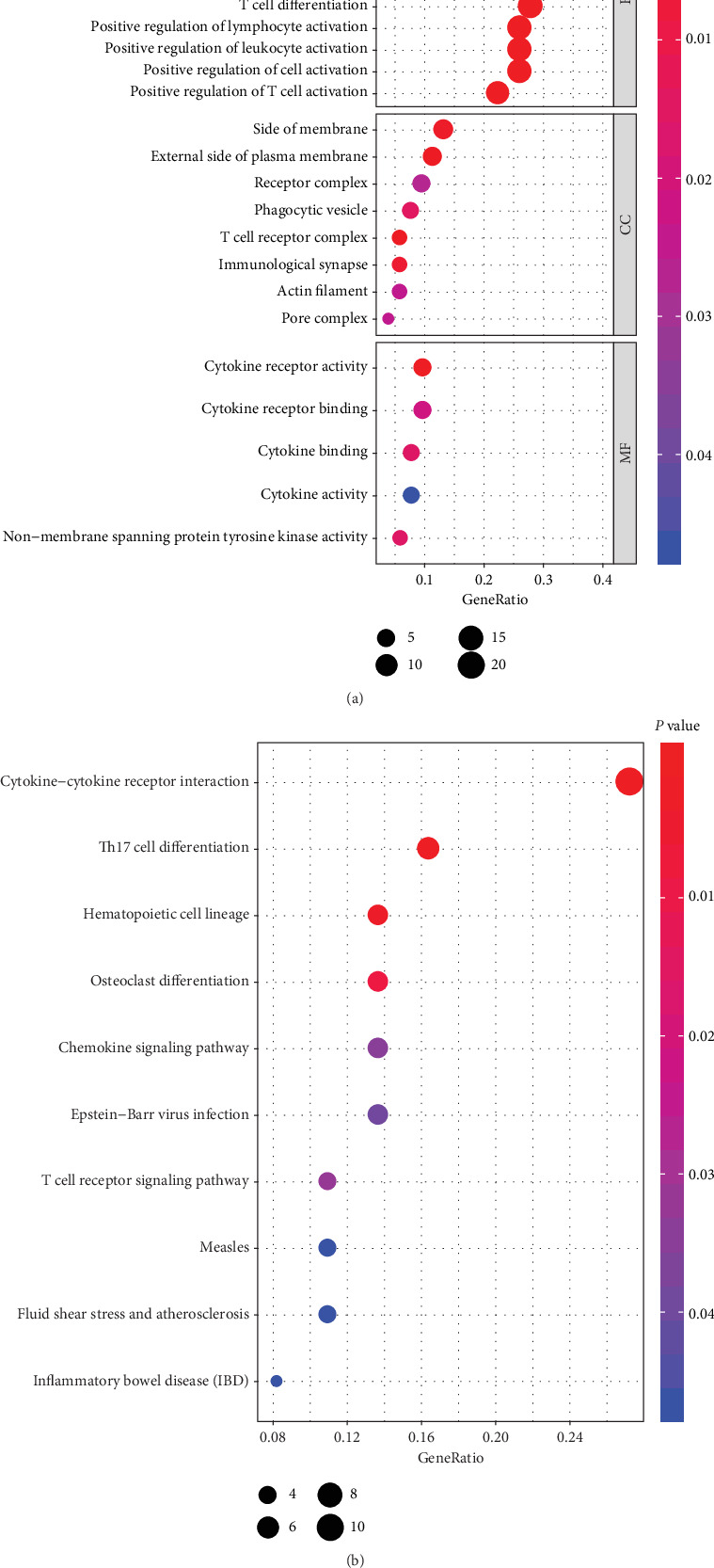
Functional enrichment analysis results of immune genes. (a) GO term enrichment results. (b) KEGG pathway analysis.

**Figure 7:**
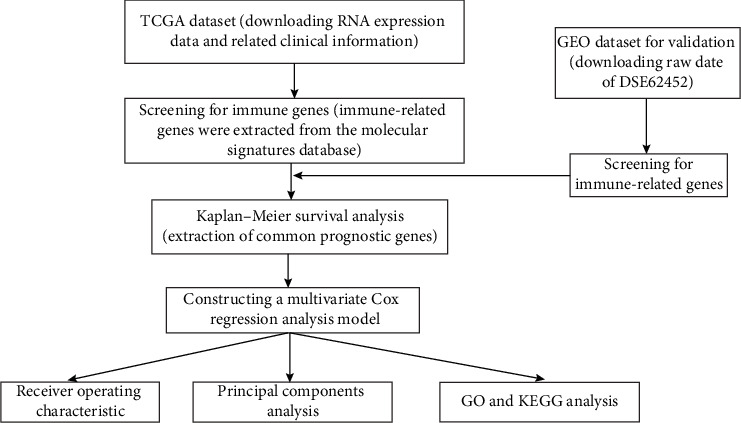
Flow chart of data preparation processing analysis and validation.

Immune-related genes were subjected to functional enrichment analysis by applying the cluster Profiler R package. The immune-related genes from the database from TCGA in the BP group were mainly enriched in T cell activation and regulation of leukocyte activation. The genes in the CC group were significantly enriched on the side of the membrane and the external side of the plasma membrane. The genes in the MF group were mainly enriched in cytokine receptor activity and cytokine receptor binding (*P* < 0.05; Figure 6(a)). KEGG analysis suggested that most of the immune-related gene pathways were significantly linked to cytokine-cytokine receptor interaction, Th17 cell differentiation, and hematopoietic cell lineage (*P* < 0.05; Figure 6(b)). The workflow of this study is shown in Figure 7.

## 4. Discussion

Pancreatic cancer is a common cause of death and has poor prognosis. The 5-year survival rate of patients with this disease is approximated to be less than 5% [1]. PAAD is treated with numerous strategies, including surgery, neoadjuvant therapy, chemotherapy, targeted molecular therapy, radiation therapy, and immunotherapy. However, the effects of treatment methods are limited, and novel methods for PAAD treatment should be further explored to provide patients with personalized treatment and improve their survival. With remarkable progress in bioinformatics, the mining of TCGA databases has been increasingly applied to predict cancer prognosis in many studies [15, 22, 23]. In the current study, we attempted to identify immune-related genes that contributed to the OS of patients with PAAD using a database from TCGA.

First, two datasets (TCGA and DSE62452) were collected to study the prognosis of immune-related genes in patients with PAAD. A total of 332 immune-related genes were extracted from a database from TCGA. Immune-related genes were also extracted from DSE62452 and identified by constructing a coexpression network of immune-related genes. Genes with *P* < 0.05 and |HR| >1.00 were considered to be prognostic risk genes. Then, three identified genes, CKLF, ERAP2, and EREG, were found to be associated with a high risk in the databases from TCGA and GEO. The signature of three immune-related genes was developed using a risk score method, and the patients with PAAD were divided into low- and high-risk groups based on their median risk score. The results showed that the prognosis of the patients in the high-risk group was worse than that in the low-risk group. Multivariate Cox regression analysis was conducted to determine the independent prognostic factors of PAAD. Then, the prognostic signature was comprehensively analyzed on the basis of immune-related gene expression. Kaplan-Meier analysis and the AUC comparison of the ROC curve confirmed that the three immune-related gene signatures were reliable for OS prediction. Next, a nomogram was established and integrated with a signature of three immune-related genes and clinical data, and OS was accurately predicted. The PCA results revealed that the prognostic signature of immune-related genes could clearly distinguish patients in the high-risk group from those in the low-risk group. The KEGG pathway analysis indicated that most of the immune-related genes were significantly associated with cytokine-cytokine receptor interaction, Th17 cell differentiation, and hematopoietic cell lineage. Therefore, the nomogram could be used as a progression indicator and predictor of the OS of patients with PAAD.

In our current study, three genes associated with poor prognosis of PAAD were identified: *CKLF*, *ERAP2*, and *EREG*. *CKLF* is a protein-encoding gene whose product is a cytokine. Cytokines are small proteins that play important roles in immune and inflammatory responses. The protein encoded by *CKLF* is an effective chemoattractant of neutrophils, monocytes, and lymphocytes [24, 25]. Some studies have shown that high neutrophil levels are associated with the prognosis of patients with PAAD [26, 27]. CKLF has four isoforms, designated CKLF1–4; among them, CKLF1 has the highest expression level [24]. Previous reports suggested that CKLF1 expression may play an essential role in the development of atopic dermatitis [28] and psoriasis [29]. The GO annotation related to this gene included chemokine activity. Chemokines not only participate in cancer-associated inflammation but also promote tumor development and progression [30]. CKLF1 is highly expressed in malignant ovarian cancer, providing a new basis for the clinical diagnosis and treatment of tumors [31]. Therefore, the high *CKLF* expression in PAAD might be the cause of PAAD pathogenesis and progression.

The GO annotations related to *ERAP2* included metallopeptidase and aminopeptidase activities. Human ERAP2 was initially identified as a homolog of human placental leucine aminopeptidase or insulin-regulated aminopeptidase [32]. *ERAP2* increases susceptibility to autoimmune diseases, infectious diseases, and cancer because of its genetic variability [33]. ERAP2 is associated with several immune-mediated diseases, including ankylosing spondylitis, psoriasis, and Crohn's disease [32, 34]. *ERAP2*-related pathways include class I MHC-mediated antigen processing and presentation and the innate immune system. Data from the cBioPortal website (http://www.cbioportal.org) showed that *ERAP2* is highly expressed in pancreatic cancer. *ERAP1* and *ERAP2* may be important targets that enhance T and NK cell-mediated immune responses against established cancers [35].

*EREG* is closely related to pancreatic cancer development [36]. *EREG* is a member of the epidermal growth factor (EGF) family of peptide growth factors [37]. The stimulation of the EGFR pathway also promotes tumor cell migration, adhesion, and metastasis [38]. AREG and EREG are required for autocrine EGFR signaling, indicating that EREG plays an important role in tumor progression [39]. EREG is expressed in a variety of adult tissues, and its increased expression or activity appears to promote the progression of several different human malignancies [40]. Studies have shown that EREG enhances the migration and chemotaxis ability of adipose-derived stem cells [41]. In colorectal cancer, EREG serves as a biomarker of anti-EGFR therapy [42]. The inhibition of EGFR signaling in pancreatic cancer may lead to a decrease in the growth and invasion of pancreatic tumors [43]. Previous studies revealed that EREG is upregulated in pancreatic cancer and stimulates the growth of pancreatic cancer cells [36]. Thus, our research method could be reliably used to identify prognosis-related genes for PAAD.

These risk genes could be potential molecular targets for PAAD treatment. The results of gene screening demonstrated that *EREG* was associated with the prognosis of pancreatic cancer, so our approach could be used to accurately screen prognostic genes. For the newly discovered *CKLF* and *ERAP2*, further experiments are needed to determine whether they are related to the prognosis of PAAD and verify our results. Cancer immunotherapy, which relies on the immune system to eliminate primary tumors, has shown unique advantages for cancer treatment [44]. The 2018 Nobel Prize in physiology or medicine was awarded to pioneers in the field of cancer immunotherapy, which has been a tremendously successful area of work [45]. Tumor immunotherapy has been widely explored in various fields, such as nanotechnology-enhanced immunotherapy [46, 47].

With the continuous improvement of immunotherapy technology, the indication of PAAD immunotherapy needs to be further studied. Immunotherapy is recommended for patients with pancreatic cancer with MSI/MMR molecular characteristics and distant metastasis [48]. Most patients diagnosed with PAAD for the first time are in advanced stages, missed the best time for surgical treatment, or the patient cannot tolerate surgery. PAAD is composed of dense connective tissue and highly infiltrating immune cells, which is very easy to induce chemotherapy resistance [49]. After treatment with conventional chemotherapy drugs, the patient's survival status and quality of life did not improve significantly. Therefore, in order to obtain a better prognosis for patients, immunotherapy can be selected according to the immune microenvironment of PAAD. Some studies have shown that the establishment of an “immune score” system for expressing pancreatic cancer could be used to assess the degree of immune cell infiltration in the tumor immune microenvironment [50]. Improving our understanding of how PAAD immune and stromal components interact and the tumor microenvironment can help improve our immunotherapy [51, 52]. Future strategies using immunotherapy to treat pancreatic cancer include changing immune checkpoint inhibitors from monotherapy to combination therapy and combining immunotherapy with chemotherapy, radiation therapy, and targeted therapy [53]. Due to the obvious heterogeneity among individuals with PAAD, the uses of immunotherapy will be based on the results of genetic testing, so that a personalized treatment plan can be implemented to improve the efficacy of the treatment [54]. In the future, gene sequencing technology is expected to detect and identify high-risk immune genes of PAAD and provide new directions for precise immunotherapy of PAAD.

Immune-related genes are associated with survival and may be used as biomarkers to assess the suitability of various immunotherapies. Our immune prognosis gene signature provided a novel idea and methods related to the molecular mechanism and prognosis prediction of PAAD. This signature might help solve the problem of poor immunogenicity in PAAD and improve the effectiveness and safety of cancer immunotherapy. Fortunately, because of remarkable advancements in whole-genome sequencing technology and bioinformatics, some high-throughput tumor databases have been generated and can be used for public academic research. The pathways involved in the prognosis of PAAD can also be further studied. The risk gene signaling pathway of PAAD can be inhibited to achieve the purpose of immunotherapy. The nomogram also indicated that the risk score of our prognosis genes could reliably predict the OS of patients with PAAD. However, our study had certain limitations compared with previous studies. First, our clinical information was mainly obtained from databases from TCGA and GEO. Some patients' clinical information was incomplete, and detailed data on patient prognosis were unavailable. Second, a signature of three immune-related genes was generated, and a normal tissue control was not prepared. As such, our data were not convincing enough to establish a prognostic model. The expression level of prognosis-related genes and their molecular mechanisms in the pathogenesis and progression of PAAD should be further explored experimentally. The screened genes could be verified through real-time PCR and Western blot.

## 5. Conclusions

In summary, we constructed immune-related gene coexpression networks and identified a signature of three immune-related genes that had prognostic value for patients with PAAD. The prognosis for patients in the high-risk group was worse than that in the low-risk group. Further research on these immune-related genes would help fully understand the potential links of the immune system and responses to the prognosis of PAAD. The prognostic nomogram could reliably predict the OS of patients with PAAD and might be used as a guide for the diagnosis and immunotherapy of PAAD. However, our results were not further verified experimentally because of the limitations of this investigation, so more in-depth studies should be conducted to reveal the relationship between the prognosis of PAAD and immune-related genes.

## Figures and Tables

**Table 1:** Clinical and pathologic characteristics of PAAD patients and prognostic analysis.

Variables	Events	Total (*n* = 142)	MST (days)	HR (95% CI)	*P* value
Age (years)					0.344
≤60	22	45	593	1	
>60	56	97	568	1.270 (0.774-2.086)	
Gender					0.441
Female	40	67	532	1	
Male	38	75	614	0.838 (0.536-1.312)	
Alcohol history^a^					0.742
No	27	49	532	1	
Yes	43	81	598	1.084 (0.670-1.756)	
Tumor stage^b^					0.660
I	5	13	598	1	
II	70	122	568	1.292 (0.519-3.221)	
III + IV	3	6	545	1.086 (0.252-4.667)	
Histologic grade^c^					0.140
G1	8	19	627	1	
G2	44	82	603	1.425 (0.648-3.135)	
G3 + G4	26	40	473	1.632 (0.734-3.628)	
Radical resection^d^					0.014
R0	43	81	627	1	
R1 + R2 + RX	32	53	394	1.812 (1.126-2.916)	
Risk score					0.009
Low	33	71	691	1	
High	45	71	486	1.852 (1.165-2.944)	

Abbreviations: PAAD: pancreatic adenocarcinoma; MST: media survival time; HR: hazard ratio; CI: confidence interval.

Notes: ^a^Infomation of alcohol history were not acquired in 12 patients; ^b^infomation of tumor stage were not acquired in 1 patient; ^c^infomation of histologic grade were not acquired in 1 patient; ^d^infomation of radical resection were not acquired in 8 patients.

## Data Availability

The data used to support the findings of this study are available from the corresponding author upon request.

## Abbreviations

PAAD: Pancreatic adenocarcinoma
TCGA: The Cancer genome atlas
GEO: Gene expression omnibus
MSigDB: Molecular signatures database
GSEA: Gene set enrichment analysis
HR: Hazard ratio
CI: Confidence intervals
OS: Overall survival
ROC: Receiver operating characteristic
AUC: The area under curve
PCA: Principal components analysis
GO: Gene ontology
KEGG: Kyoto encyclopedia of genes and genomes
BP: Biological process
CC: Cellular component
MF: Molecular function.

## Supplementary Materials

Supplementary Materials Table S1: the univariate survival analysis results of the TCGA and GEO cohort.
